# *PTEN* Hamartoma Tumor Syndrome: A Clinical Overview

**DOI:** 10.3390/cancers11060844

**Published:** 2019-06-18

**Authors:** Robert Pilarski

**Affiliations:** Division of Human Genetics, Department of Internal Medicine and Comprehensive Cancer Center, The Ohio State University, Columbus, OH 43221, USA; Robert.pilarski@osumc.edu; Tel.: +1-614-293-7774; Fax: +1-614-293-2314

**Keywords:** PTEN, PHTS, *PTEN* harmartoma tumor syndrome, cowden syndrome, Bannayan-Ruvalcaba-Riley syndrome

## Abstract

The phosphatase and tensin homolog (*PTEN)* hamartoma tumor syndrome (PHTS) is a grouping of related genetic disorders that has been linked to germline mutations in the *PTEN* gene. These disorders include Cowden syndrome (CS), Bannayan–Riley–Ruvalcaba syndrome, adult Lhermitte–Duclos disease, and autism spectrum disorders associated with macrocephaly. The majority of the clinical information available on PHTS, however, is related to individuals diagnosed with CS. There is still much to be learned about this disorder, since diagnostic criteria for CS were only established in 1996, before the identification of the *PTEN* gene, and were based primarily on features seen in cases reported in the existing literature. More recently, however, data from several large series of patients have shown that a number of the clinical features associated with *PTEN* mutations are either more or less common than previously reported. In addition, we now know that only about 30–35% of patients meeting clinical diagnostic criteria for Cowden syndrome actually have a detectable *PTEN* mutation. Thus, our understanding of *PTEN*-related diseases and their management has evolved significantly over time. The United States National Comprehensive Cancer Network (NCCN) has produced and regularly updates practice guidelines which include clinical diagnostic criteria as well as guidelines for *PTEN* testing and management of patients with mutations. This review will summarize the overall literature on PHTS as well as recent findings which are broadening our understanding of this set of disorders.

## 1. Introduction

*PTEN* hamartoma tumor syndrome (PHTS) refers to a spectrum of disorders that have been linked to germline mutations in the *PTEN* gene. These include Cowden syndrome, Bannayan-Riley-Ruvalcaba syndrome, adult Lhermitte–Duclos disease, and autism spectrum disorders associated with macrocephaly. The majority of the published data, however, address patients with Cowden syndrome, and risks must be extrapolated to the other conditions. To date there is insufficient evidence of a correlation between specific *PTEN* mutations and clinical presentations.

Cowden syndrome (CS) is a rare, multi-system disease that was first described in one family in 1963 [1]. It entails increased risks for malignancies of a number of organs (particularly breast, thyroid and endometrium) and benign overgrowth of a number of tissues (skin, colon, thyroid, etc.). Germline *PTEN* mutations were first reported in individuals with CS in 1997 [2,3].

Bannayan-Riley-Ruvalcaba syndrome (BRRS) is a rare pediatric disorder that was initially felt to be distinct from CS. The most common clinical features include macrocephaly, hamartomatous intestinal polyps, lipomas, and pigmented macules on the penis. Other reported features include developmental delay, vascular anomalies, large birth weight, and joint hyperextensibility [4]. BRRS has been shown to be allelic to CS, with approximately 60% of patients having *PTEN* mutations [5,6]. No other genes are known to cause BRRS. However, there are relatively few data on the clinical features of BRRS patients with documented *PTEN* mutations [7].

There is broad variability in the clinical presentation of patients with *PTEN* mutations, even between patients with the same mutation and patients in the same family. Indeed, families have been reported in which one or more individuals met CS diagnostic criteria while others met BRRS criteria [5,8,9,10]. While a clear genotype/phenotype correlation has not been shown with *PTEN*, there is some evidence that mutations leading to stable but inactive proteins produce a more severe phenotype than mutations leading to proteins with partially retained function [11]. Spinelli et al. reported that all seven autism-associated mutations that they studied led to proteins that retained the ability to suppress AKT signaling [12]. Studies in mice suggest that tumor presentation is dependent on the genetic background of the animal, but the implications for humans with *PTEN* mutations are unclear [13].

Consensus diagnostic criteria for CS were initially established in 1996 by an international research consortium. This was prior to identification of the CS gene and the criteria were based upon early clinical experience and compilations of cases published in the literature, with their inherent ascertainment biases [14]. The single largest patient series in any of these reports comprised 21 patients [15]. It was not until 2013 that an evidence-based review led to a significant revision of the diagnostic criteria (summarized in Table 1), which were then adopted by the U.S. National Comprehensive Cancer Network [16,17].

It was initially reported, based on small series, that *PTEN* mutations were found in 80% of patients with CS [3,18]. More recently, in much larger cohorts, *PTEN* mutations have been found in only 30–35% of patients meeting consortium diagnostic criteria and in 23/42 (55%) patients with a clinical diagnosis of BRRS [6,19]. Importantly, 63/172 (37%) patients with mutations in one of these studies did not meet diagnostic criteria for either CS or BRRS [6]. It is estimated that at least 11%, and perhaps as many as 48%, of patients have de novo mutations [20].

The National Comprehensive Cancer Network (NCCN) has established criteria for when *PTEN* testing is indicated based on the clinical features present in a patient, as well as management and screening recommendations for individuals who are found to have a *PTEN* mutation [16]. As noted above, they have also adopted revised clinical diagnostic criteria since patient management is sometimes based on a clinical diagnosis alone if either testing is not possible or it is done but no mutation is found.

This paper reviews the current understanding of the clinical features that are most clearly associated with *PTEN* mutations.

## 2. Cancer/Tumor Risks

The risks for breast, uterine, thyroid, and colon cancer and Lhermitte-Duclos disease are clearly increased in individuals with *PTEN* mutations. The magnitude of these risks, however, is less certain. The association of *PTEN* mutations with other tumor types is less clear. Each of these is discussed further below.

### 2.1. Brain Tumors

Lhermitte-Duclos disease (LDD, dysplastic gangliocytoma of the cerebellum) is a rare, slow growing hamartoma which is usually diagnosed when patients are in their twenties or thirties. While it appears to be clearly associated with *PTEN* mutations, the incidence of LDD in patients with CS is unknown [21]. A prevalence of 1.8% (3/172) was found in patients undergoing clinical *PTEN* testing [6], of 6% (18/290) in patients in a research cohort [19], and of 15% in cases reported in the literature. [22] Wei et al. found that five of seven adults with LDD had features of PHTS, although *PTEN* testing was not done [23]. Based on a small study of unselected LDD tumors from 15 adults and three children, it appears that adult-onset LDD is more strongly associated with *PTEN* mutations than childhood-onset LDD [24]. In another review of 14 children with LDD, three had clinical diagnoses of CS, eight had no signs of the disease, and three had insufficient information to determine a diagnosis [25].

While various case reports have suggested that a variety of other brain tumors are linked to *PTEN* mutations, the true spectrum and frequency of brain lesions is unknown since brain imaging is rarely done on asymptomatic individuals. Only one report of brain magnetic resonance imaging (MRI) in asymptomatic CS patients has been published [26]. In this study, seven of 20 patients (mean age 42 years) had brain abnormalities (including three patients with LDD): six had venous and cavernous angiomas, and one had a meningioma. Although multiple cases of meningioma have now been reported in CS (reviewed in Lok et al. [26] and Yabubov et al. [27]), no systematic study has been done and their prevalence in PHTS is unknown. Lok et al. found hamartomatous vascular malformations in almost a third of their patients [26].

### 2.2. Breast Cancer

Although initially not regarded as being part of the syndrome, breast cancer has long been recognized as the most common CS malignancy. The age of onset appears to be young (average age 38–50 years), and the lifetime risk is typically quoted to be 25–50% [6,15,19,28,29]. Several more recent reports have projected the lifetime risk of breast cancer to be 77–85% in several cohorts [22,30,31]. Selection bias was a significant issue in each of these studies, however, as lifetime risks were projected based on the prevalence of breast cancer in the patients who presented for testing (which typically includes those most likely to have cancer). Thus, while breast cancer risk is clearly increased in individuals with *PTEN* mutations, there remains debate as to the degree of that risk. Three males with *PTEN* mutations and breast cancer have been reported [18,32,33], but male breast cancer was not seen in the two largest cohorts reported to date, and a possible association with male breast cancer remains unproven [6,19].

### 2.3. Gastrointestinal Cancer

Although it was originally felt that *PTEN* mutations did not increase the risk for colon cancer [34], more recent data has shown otherwise. Several studies have shown the prevalence of colon cancer (often early-onset) in several *PTEN* mutation-carrier cohorts to be 9–13% [35,36]. Two later publications predicted lifetime colon cancer risks of 9% (Confidence Interval (CI) 3.8–14.1%) and 16% (CI 8–24%), although both were limited by ascertainment biases common in PHTS studies [22,31]. A small study from the Mayo Clinic found colon cancer in two of 13 CS patients [37].

There are at least four reports of gastric cancer in CS patients, but at this point it is not considered part of the PHTS spectrum [34,38,39,40].

### 2.4. Endometrial Cancer

Endometrial cancer was found in 14.1% and 7.6% of female *PTEN* mutation carriers in two large research series [6,19], and in 17% of clinically tested adult females in another cohort, with the greatest increase in women under age 50 [6]. As noted above with breast cancer, three studies on cancer risks in *PTEN* mutation carriers have projected an increased lifetime risk of endometrial cancer ranging from 19 to 28% at age 70, but each suffers from ascertainment bias [22,30,31]. Among 54 families in another study with uterine and breast cancers and at least one other *PTEN*-related cancer, no *PTEN* mutations were identified [41]. In contrast, 7% of a cohort of endometrial cancer patients with features of Cowden syndrome had *PTEN* mutations in another report, although it is not possible to tell from the data how many of these met CS/PHTS diagnostic criteria [42]. Among 381 endometrial cancer patients who underwent screening for Lynch syndrome, one had a *PTEN* mutation [43]. Among unselected case of endometrial cancer inherited *PTEN* mutations are quite rare [44].

### 2.5. Melanoma

Various case reports have suggested that melanoma risk may be increased in individuals with *PTEN* mutations [45,46,47,48]. However it was found in only about 1% (2/172) of patients with mutations in one recent cohort, although the mean age of the cohort was only 30 years [6]. More recently, one group projected a 6% lifetime risk for melanoma (based on nine cases of melanoma reported among 368 patients with mutations) while a second projected standardized incidence ratios (SIRs) of 28.3 in women and 39.4 in men, (based on nine cases among 154 mutation-positive patients) [30,31]. The lifetime risk of developing melanoma in the United States is almost 2.8% [49]. Given this limited evidence, melanoma is not included as a diagnostic criterion for PHTS and more data are needed regarding its association with *PTEN*.

### 2.6. Thyroid Cancer

Historically, the lifetime risk of thyroid cancer in *PTEN* mutation carriers was estimated to be 3–10% [50]. However, recent analyses of two cohorts of *PTEN* mutation carriers (with the same ascertainment biases noted above for other cancers) have projected lifetime risks of 35–38% [30,31]. Whether these are significant overestimates remains to be determined. Papillary (including follicular variant) thyroid cancer represents 56–60%, while follicular pathology accounts for 25–45% of reported cancers [51,52]. Medullary thyroid cancer is not felt to be associated with *PTEN* mutations. 

The prevalence of *PTEN* mutations in unselected individuals presenting with differentiated thyroid cancer appears to be low, with only two mutations found in 259 individuals in one study. Both patients had follicular thyroid cancer and other features of PHTS such as macrocephaly. Among follicular thyroid cancer cases 2/42 harbored *PTEN* mutations [53]. Another study found no *PTEN* mutations in women presenting with both primary breast and thyroid cancers [54]. 

### 2.7. Renal Cell Carcinoma

The risk for renal cell carcinoma (RCC) was initially suggested to be increased in CS based on several case reports [55,56]. More recent case series of patients with *PTEN* mutations reported RCC in 5% of 107 clinically tested adults and 3–6.7% of adults who had research testing [6,19]. Two studies recently projected the lifetime risk for RCC in individuals with *PTEN* mutations to be approximately 34%, although these were based on small numbers of cases and may be an overestimate reflecting ascertainment bias [30,31]. In another series, four of 24 (16.7%) patients with CS were found to have a total of five RCC, although none had a family history of RCC [57]. Loss of heterozygosity for *PTEN* was shown for four of the five tumors. Among eight PHTS patients with RCC, Mester et al. found papillary histology in six and chromophobe histology in two [58]. Immunohistochemistry showed complete loss of *PTEN* staining in all six papillary tumors and patchy positivity in one of the chromophobe tumors. Germline *PTEN* mutations are rare among individuals with non-syndromic hereditary RCC [57].

## 3. Benign Disease

As noted above, individuals with germline *PTEN* mutations are at risk for a range on non-malignant disease. The actual prevalence of most of these in PHTS patients, however, is difficult to determine.

### 3.1. Brain Lesions

There are limited data on the prevalence of developmental delay and mental retardation (MR/DD) among individuals with *PTEN* mutations. The prevalence does seem to be increased over the general population rate of 3%, however, with MR/DD reported in 12% [29] and 15–20% [59] of cases in the literature, and in 17% of 110 mutation-positive patients who underwent clinical testing [6]. It has been reported that 15–20% of patients with BRRS have mental retardation and an additional 50% may have motor and speech delays but normal adult intelligence [59]. There is a broad range of manifestation, from normal intelligence (in most), to mild-significant developmental delays/MR, to autism spectrum disorders.

Based on cases reported in the literature, macrocephaly (defined as a head circumference greater than the 97th percentile) was initially reported to be present in 40% of CS patients. It has since been found in 80–100% of patients with *PTEN* mutations whose head circumference had been measured, [6,19,21,60,61]. Macrocephaly is also seen in the majority of BRRS patients, but it is also one of the few diagnostic criteria for the condition so it would be expected to be frequently seen [59]. Hansen-Kiss et al. found macrocephaly in 46 of 47 (98%) pediatric patients with *PTEN* mutations in their study [62]. Vanderver et al. reported the brain MRI findings (including enlarged perivascular spaces and multifocal periventricular white matter abnormalities) in a cohort of 23 patients with *PTEN* mutations and macrocephaly [63], while Ciaccio et al. found various brain abnormalities in 75% of pediatric *PTEN* mutation carriers who had imaging [64] and Hansen-Kiss et al. found a range of abnormalities in 53% of 17 children with *PTEN* mutations who had brain imaging [62]. Balci et al. reported on nine patients with *PTEN* mutations and white matter changes on brain MRI who presented with a range of developmental phenotypes [65].

Germline mutations in *PTEN* also cause a subset of patients with both autism spectrum disorders (ASD) and macrocephaly, with patients with the largest head sizes being more likely to have a mutation [66,67,68,69,70,71,72,73]. In these reports *PTEN* mutations have been found in 1–27% of patients with ASD and macrocephaly, even in absence of other personal or family history suggestive of PHTS. Some evidence suggests that *PTEN* mutations retaining partial function may lead to milder phenotypes such as these [11,12,74].

### 3.2. Gastrointestinal Disease

Despite initial reports that colonic polyps were found in 40% of patients with CS, recent data has shown that polyps are found in up to 95% of adults with *PTEN* mutations who have undergone colonoscopy [36,37]. Polyp numbers can be few or numerous (even hundreds), may sometimes occur in childhood, and are distributed throughout the colon. The range of polyp types seen includes adenomas, ganglioneuromas, hamartomas, inflammatory polyps, leiomyomas, lipomas, and lymphoid polyps [36,50,75,76]. Hyperplastic polyps have also been seen, but they are common in the general population and have not been observed in all studies so the association with *PTEN* is unclear [36,77]. The majority of CS patients have multiple synchronous histologic types at colonoscopy. Among 603 patients with five or more colon polyps (including at least one hamartomatous or hyperplastic polyp), Ngeow et al. found germline mutations in known non-adenomatous polyposis genes in 13%, including *PTEN* mutations in 2.2% [78]. Patients with ganglioneuromas, intramucosal lipomas, and/or those with three or more polyp types were more likely to harbor *PTEN* mutations [76,79]. Shaco-Levy et al. described a similar range of histologies among the polyps seen in 13 patients with CS [80]. Henderson et al. suggested that eosinophilic gastrointestinal disorders may be more common in children with *PTEN* mutations [81].

The esophagus in PHTS is characterized by glycogenic acanthosis [82]. One or several such lesions may occasionally be observed in unaffected individuals, but numerous and diffuse lesions were seen in the majority (80% or more) of CS patients in one study [77]. Although one group has recommended that diffuse esophageal glycogenic acanthosis combined with colonic polyposis should be considered pathognomonic for CS, this has not been incorporated into the NCCN diagnostic criteria [83].

Upper gastrointestinal lesions are also seen in PHTS, with multiple hamartomatous polyps in the stomach, duodenum, and small bowel reported [34,36,77,82]. These included adenomas, hamartomas, and inflammatory and hyperplastic polyps (different from colonic hyperplastic polyps), as well as ganglioneuromas.

### 3.3. Skin Lesions

A number of dermatologic lesions are common in PHTS. While initial reports (published at a time when dermatologic features were required to make a clinical diagnosis of Cowden syndrome) suggesting dermatologic lesions are present in almost 100% of CS patients are likely overestimates, it is clear that they are a common finding in individuals with *PTEN* mutations.

Among these, multiple trichilemmomas are most strongly indicative of a *PTEN* mutation. These can be seen on the face including the eyes, mouth, nose, and forehead [15,48,84,85,86,87], as well as the neck, axillae, and hand [88,89]. The prevalence of trichilemmomas in individuals with a confirmed *PTEN* mutation ranges from 6 to 25% in large case series, with some patients presenting under 18 years of age [3,6,19,36]. Trichilemmomas are clinically indistinguishable from trichoepitheliomas, fibrofolliculomas, and other benign skin lesions and thus require histopathology to confirm a diagnosis [85,86,90,91].

Acral keratoses, located on the palmoplantar surfaces and dorsal hands/feet, have been repeatedly reported in both children and adults with PHTS [15,29,48,88,92,93,94]. Acral keratoses have been noted in both pediatric and adult populations of *PTEN* mutation carriers, but further studies are needed to define the age of onset and penetrance [19,36,93,95]. Some case reports observed keratoses appearing on non-acral sites as well [15,48]. At this time further studies are needed to determine the penetrance and age of onset of acral keratoses in PHTS, as well as to determine if they are more common in non-acral sites as well.

Sclerotic fibromas of the skin, which are rare in the general population, have been reported in a number of case series of patients with a clinical diagnoses of Cowden syndrome prior to the identification of the *PTEN* gene, and in individual case reports of patients with mutations [15,96,97,98]. Among clinically diagnosed Cowden syndrome patients the prevalence of oral fibromas ranges between 14 and 76% [15,29]. Although more common in the general population than sclerotic fibromas, the incidence of oral fibromas in the general population is not well defined. Further studies are needed in individuals with *PTEN* mutations to characterize the prevalence and natural history of both skin and oral fibromas. 

Lipomas are included in the clinical diagnostic criteria for both BRRS and CS. In a prospective cohort of 43 clinically and/or genetically diagnosed BRRS cases compared to 37 Cowden syndrome families, the features of lipomas and vascular abnormalities were more commonly observed in BRRS or BRRS/Cowden syndrome overlap families [5]. The presence of multiple lipomas in an individual in the general population is rare but this has been reported in several small and large case series of individuals with a *PTEN* mutation. [6,15,36,48,55,88,91,93,99,100,101,102,103].

Mucocutaneous neuromas (hamartoma of the peripheral nerve sheath) have been reported on the face, hands, shins, and back in numerous patients with PHTS, with Starink et al. (reporting before the identification of the *PTEN* gene) finding them in more than half of their clinically-diagnosed patients by age 18 [15,48,101,104]. At this time further studies are needed to determine the prevalence and clinical features of neuromas seen in PHTS.

Oral papillomas, seen on the lips, tongue, buccal mucosa, and gingivae, are common in PHTS, although the florid papillomatosis shown in some publications is rare [15,29,48,84,88,89,91,92,103,105,106]. They typically present by the second decade of life and are usually asymptomatic, in contrast to neuromas [15,84,88,89,93,101,104]. 

While benign pigmentation of the genitals is reported in up to 15% of males in the general population [107], their prevalence is clearly increased in males with *PTEN* mutations. In one large cohort, 48% of males with either a clinical diagnosis or a *PTEN* mutation had significant penile freckling, while in another study, 53% of male *PTEN* mutation carriers had this finding [6,19]. Hansen-Kiss et al. found penile freckling in 12/29 (41%) of boys with *PTEN* mutations, the youngest at 15 months of age [62].

Vascular (venous or arterial) anomalies are reportedly common in BRRS and CS, particularly in patients with a BRRS diagnosis. A number of case reports support the association of arteriovenous malformations in clinically diagnosed BRRS patients [108,109,110,111]. Among 20 CS patients undergoing brain MRI, Lok et al. noted venous angiomas in five and cavernous angiomas in two [26]. Tan et al. reported on the vascular anomalies seen in a cohort of 23 patients with *PTEN* mutations, while Kurek et al., reporting on a cohort of patients with known or suspected *PTEN* mutations, noted a distinctive lesions they designated as a *PTEN* hamartoma of soft tissue [21,112]. Smaller case series have specifically reported hemangiomas and cavernous hemangiomas in both children and adults with *PTEN* mutations [62,94,101,102]. Pimpalwar et al. reported on the natural history and management of fast flow vascular anomalies in four patients [113]. In the general population, certain subtypes of hemangioma are sporadic and common in children or adults [114]. Thus, further studies are needed on the prevalence of vascular lesions in PHTS.

Testicular lipomas have only been noted in PHTS since 2003, being first reported in a 39 year-old male with multiple fat-containing testicular hamartomas [115]. Since then, Woodhouse et al. reported findings from testicular ultrasound in a series of eight males (ages 16 to 58) with documented *PTEN* mutations, finding multiple, bilateral hyperechoic lesions in all but the youngest patient [116,117]. Biopsies from four of the patients confirmed lipomas. Subsequently, multiple other cases of testicular lipomas in males with CS or BRRS have been reported [118,119,120,121,122]. Testicular lipomas are very rare in the general population in absence of testicular neoplasia [117], with Harper et al. finding only one case in a Medline search from 1970 to 2001 [123]. Although their prevalence in males with *PTEN* mutations is unknown, the presence of multiple testicular lipomas has been added to the clinical diagnostic criteria for CS/PHTS [17].

### 3.4. Thyroid Disease 

Benign thyroid disease is common in PHTS, with thyroid nodules, adenoma, or goiter reported in 30–68% of adults and 2–14% of children with *PTEN* mutations [6,19]. Tan et al. reported a prevalence of 3–21% specifically for Hashimoto thyroiditis in individuals with *PTEN* mutations [19]. Hall et al. reviewed the thyroid findings in 181 CS patients reported in the literature, with their inherent ascertainment and publication biases, and found thyroid disease reported in 53% of patients, with 80% of these requiring surgical management [124]. It should be noted that almost 38% of these patients did not have genetic testing to confirm the presence of a *PTEN* mutation. Thyroid lesions are very common in the general population, which complicates efforts to determine their prevalence in PHTS. The prevalence of thyroid nodules in the general population ranges from 2 to 6% on physical exam to 19–35% on ultrasound to as high as 65% in autopsy series [125]. Multinodular goiter is found in about 4% of the population, [126] while Hashimoto’s thyroiditis occurs in 2% [127].

Hansen-Kiss et al. found abnormal thyroid imaging (cysts, nodules and goiter) in 26% of their series of 47 pediatric patients with *PTEN* mutations [62]. Plamper, reporting on patients seen in a pediatric endocrine clinic, found thyroid abnormalities in 12 of 16 (75%) children with *PTEN* mutations in their series, with eight of them undergoing thyroidectomy [128]. Pathology varied from nodular goiter, follicular adenoma and autoimmune disease to papillary microcarcinoma in a six-year-old boy and follicular carcinoma in a 13-year-old girl. 

There are limited data on the clinical features and pathology of thyroid nodules in *PTEN* mutation carriers. One review of thyroidectomy specimens from 20 individuals found that all had multiple findings, most commonly multinodular goiter (75%), which was often seen along with thyroiditis (55%) [52]. Another study of the thyroid pathology from 25 individuals with *PTEN* mutations had similar findings [129]. A pediatric study found thyroid nodules at as young as age 6 [130]. It is important to note the ascertainment biases in these studies, however, since most were evaluating individuals who presented with significant and overt thyroid disease, and the frequency and clinical presentation in unselected mutation carriers is unknown.

### 3.5. Other Benign Diseases

Other benign lesions such as fibrocystic and other benign breast diseases, uterine fibroids, and genito-urinary malformations were initially included in the clinical diagnostic criteria for CS. However, there is insufficient evidence to show that they are more common among individuals with *PTEN* mutations than in the general population (in which fibrocystic breast disease and uterine fibroids in particular are very common), and they have been dropped from the clinical diagnostic criteria [16,17]. While a range of other breast lesions (sometimes quite complex) have been reported in case reports or small series of women with clinical or molecular diagnoses of Cowden syndrome, there are no good data on unselected patient populations [131,132]. 

While more data are needed, a growing number of reports suggest possible immune system dysregulation in some individuals with germline *PTEN* mutations, as shown in mouse models [133,134,135,136,137]. In addition, there is evidence for the role of *PTEN* in metabolic regulation and for a form of constitutive insulin sensitivity in some individuals with *PTEN* mutations [138,139,140,141].

## 4. Conclusions

As has been shown, our understanding of the clinical and molecular features of *PTEN* hamartoma tumor syndrome continues to evolve. As with many rare conditions, it has been difficult to obtain data on substantial numbers of patients with PHTS. The larger patient cohorts that have been assembled suffer from significant ascertainment bias since most of the patients were tested specifically because they presented with significant clinical features of PHTS. To date, no large cohort study has been published on mutation carriers who are relatives of the probands who were initially tested (which would help reduce ascertainment bias). Unfortunately, it is unlikely that such a cohort could be assembled, and thus we must rely on the data that are available. In recent years, various publications have improved on our understanding of the prevalence of *PTEN* mutations, macrocephaly, and colon disease in particular in PHTS patients, and have added testicular lipomatosis and possibly immunologic problems to the PHTS phenotypic spectrum. At the same time, we have seen that the data do not support the inclusion of previously long-held features such as fibrocystic breast, uterine fibroid, and genito-urinary malformations. Over time we can expect to see further clarification of the phenotypes of this fascinating set of disorders.

## Figures and Tables

**Table 1:** Revised *PTEN* hamartoma tumor syndrome (PHTS) diagnostic criteria.

Revised *PTEN* Hamartoma Tumor Syndrome (PHTS) Clinical Diagnostic Criteria [16,17]
**Operational Diagnosis in an Individual (either of the following):**
(1) Three or more major criteria, but one must include macrocephaly, Lhermitte–Duclos disease, or gastrointestinal hamartomas; OR
(2) Two major and three minor criteria;
**Operational Diagnosis in a Family where One Individual Meets Revised PHTS Clinical Diagnostic Criteria or Has a *PTEN* Mutation:**
(1) Any two major criteria with or without minor criteria; OR
(2) One major and two minor criteria; OR
(3) Three minor criteria
**Major Criteria**
Breast cancer
Endometrial cancer (epithelial)
Thyroid cancer (follicular)
Gastrointestinal hamartomas (including ganglioneuromas, but excluding hyperplastic polyps) (≥3)
Lhermitte–Duclos disease (LDD), adult
Macrocephaly (≥97 percentile)
Macular pigmentation of the glans penis
Multiple mucocutaneous lesions (any of the following):
Multiple trichilemmomas (≥3), at least one biopsy proven
Acral keratoses (≥3 palmoplantar keratotic pits and/or acral hyperkeratotic papules)
Mucocutaneous neuromas (≥3)
Oral papillomas (particularly on tongue and gingiva), multiple (≥3) OR biopsy-proven OR dermatologist-diagnosed
**Minor Criteria**
Autism spectrum disorder
Colon cancer
Esophageal glycogenic acanthosis (≥3)
Lipomas (≥3)
Mental retardation (i.e., Intelligence Quotient (IQ) ≤ 75)
Renal cell carcinoma
Testicular lipomatosis
Thyroid cancer (papillary or follicular variant of papillary)
Thyroid structural lesions (e.g., adenoma, multinodular goiter)
Vascular anomalies (including multiple intracranial developmental venous anomalies)
